# Selective Anti-Proliferation of HER2-Positive Breast Cancer Cells by Anthocyanins Identified by High-Throughput Screening

**DOI:** 10.1371/journal.pone.0081586

**Published:** 2013-12-03

**Authors:** Weihua Liu, Jinmei Xu, Shaoping Wu, Yilun Liu, Xiaoping Yu, Juan Chen, Xi Tang, Zhi Wang, Xiaohu Zhu, Xin Li

**Affiliations:** 1 Department of Scientific Research, First Affiliated Hospital of Chengdu Medical College, Chengdu, Sichuan Province, China; 2 Department of Public Health, The Chengdu Medical College, Chengdu, Sichuan Province, China; 3 Department of Medical Image, First Affiliated Hospital of Chengdu Medical College, Chengdu, Sichuan Province, China; 4 Administrative Office of Chengdu Medical College, The Chengdu Medical College, Chengdu, Sichuan Province, China; 5 Department of Acupuncture and Moxibustion, Xindu Hospital of Traditional Chinese Medicine, Chengdu, Sichuan Province, China; Rutgers - New Jersey Medical School, United States of America

## Abstract

Overexpressed Human epidermal growth factor receptor 2 (HER2) drives the biology of 20% breast cancer and is a prediction of a poor prognosis for patients. HER2-targeted therapies significantly improve outcomes for HER2-positive patients. Traditional Chinese herbs/medicines have been used to treat breast cancer patients including HER2-positive patients in Asia for decades. Although the traditional medicines demonstrate efficacy in clinics for HER2-positive patients, the mechanism is largely unknown. In this article, we screened a 10,000 natural product library in 6 different cell lines representing breast cancer, and assessed the ability of each drug to cause cytotoxicity through a high-throughput screening approach. We have identified eight natural compounds that selectively inhibit the proliferation of HER2-positive cells. Two of the hit compounds, peonidin-3-glucoside and cyaniding-3-glucoside, are both extracts from black rice. They inhibit the phospho-HER2 and phospho-AKT and were confirmed to induce HER2-psotive breast cancer cells apoptosis both *in vitro* and *in vivo*. Peonidin-3-glucoside and cyaniding-3-glucoside treatments significantly reduced the tumor size and volume *in vivo* compared to the control group. There is no significant difference of antitumorgenic effects between peonidin-3-glucoside and cyaniding-3-glucoside treatments.

## Introduction

Breast cancer is a serious and sometimes life-threatening disease. An estimated 232,340 new cases of invasive breast cancer are expected to be diagnosed among women in the US during 2013 according to American Cancer Society. Breast cancer has also become common to Chinese women in recent years possibly due to the change of environments, growth patterns, diet and aging. Based on the model generated by Linos et al., in 2021, the estimated breast cancer incidence rate would be 85.3 to 87.8 per 100,000 woman in China [1]. Evidently, there is a clear need for the development of new therapeutic agents.

HER2 overexpression occurs in ∼20% of patients with breast cancer and is associated with aggressive disease and decreased survival. A number of therapeutic approaches have been developed against HER2 worldwide including tyrosine kinase inhibitors, monoclonal antibodies such as Trastuzumab [2]. The mechanism has been largely studied and since then, the disease-free survival and overall survival of patients have all been improved significantly [2]–[5]. In China, there are reports of successful novel therapeutic approaches using traditional medicine for breast cancer patients [6], 7. Traditional Chinese Herbs/Medicines have developed into a mature system for more than three thousand years. Although thousands of Traditional Chinese Medicines have been proved to be effective clinically, the mechanisms of the drug actions are largely unclear. With the modern technology, researchers successfully purified and identified numerous extracts that have not been well defined before. With the rich prior human experiences, we proposed to screen a natural product library which contains 10,000 extracts against representative breast cancer cells and tried to identify compounds that selectively inhibit HER2-positive breast cancer cells.

## Materials and Methods

### Compound Library

The natural product library contains 10,000 natural products with a minimum of 98% purity confirmed by NMR and HPLC (Pharmanic, Chengdu, Sichuan, China). Briefly, compounds were extracted by supercritical CO_2_ extraction (SFE-CO_2_) and the residues after SFE-CO_2_ extraction were then refluxed with 80% ethanol and the ethanol extracts were spray-dried to obtain the extracts. Then the extracts samples were compared to the reference chemical standards purchased from the National Institute for the Control of Pharmaceutical and Biological Products (Beijing, China) by Pharmanic. The conditions of the solvent gradient elution were 8-20% (A) in 0–20 min, 20–40% (A) in 25–30 min, 40–70% (A) in 30–45 min, 70–90% (A) in 55–60 min at a flow-rate of 1.0 ml/min. Detection was conducted with different wavelengths of 230, 240, 270, 262, and 420 nm with the reference wavelength of 550 nm at room temperature. Compounds were present at 10 mmol/L in DMSO. Afatinib (BIBW2992) was gifted from the Pharmacology department of Chengdu Medical College with a >98% purity.

### Cell Culture

All cell lines were obtained from the American Type Culture Collection (ATCC) except SUM190 (**Table 1**). SUM190 cells were gifted from Chengdu Medical College bio-core facility [8]. MCF-7, MDA-MB-453 and MDA-MB-231 cells were maintained in DMEM supplemented with 2 mmol/L L-glutamine, 10% fetal bovine serum and 1% penicillin/streptomycin. BT474 cells were maintained in DMEM: Ham's F12 medium (1∶1 mixture) supplemented with 2 mmol/L L-glutamine, 5 µg/ml insulin, 10% fetal bovine serum and 1% penicillin/streptomycin. HCC1569 cells were maintained in RPMI-1640 medium supplemented with 10% fetal bovine serum and 1% penicillin/streptomycin. All cells were maintained in a 5% CO_2_ atmosphere at 37°C.

**Table 1:** Breast cancer cell lines used in HTS and their molecular classifications [4], [38].

Cell line	Classification	Immunoprofile
MCF-7	Luminal A	ER^+^, PR^+/−^, HER^2−^
BT474	Luminal B	ER^+^, PR^+/−^, HER^2+^
MDA-MB-453	Luminal	ER^−^, PR^−^, HER^2+^
SUM190	Basal	ER^−^, PR^−^, HER^2−^
MDA-MB-231	Basal B	ER^−^, PR^−^, HER^2−^
HCC1569	Basal A	ER^−^, PR^+/−^, HER^2+^

### High-throughput Screening Natural Compounds for Activity in Breast Cancer Cells

To identify compounds that might have inhibitory effect on breast cancer cell proliferation, we used a high-throughput drug screen experimental approach to assess the activity of 10,000 natural compounds. Prior to the screen, the cell viability assay was miniaturized to a 96-well, low-volume, black, flat-bottom polystyrene microplates (Corning, Shanghai, China) format to accelerate assay throughput. Cells were grown to 80% confluence, harvested and aliquoted into 96-well plates at concentrations of 1,000 cells per well in a total volume of 90 µL/well. The outer wells were inoculated with medium to minimize evaporation from the sample wells. Cells were allowed to attach overnight and 10 µl culture medium that containing either vehicle or drug at a 100 µmol/L concentration were added to give a final concentration of 10 µmol/L. Cell proliferation was evaluated using 20 µL Alamar-Blue reagent per well according to manufacturer's instructions after 72-h incubation. The plates were then incubated at 37°C for an additional 4 h and the fluorescent signal was measured. Fluorescence was measured with an excitation at 530 nm and emission at 590 nm on ZS-2 plate reader. After background subtraction, cell viability values were normalized to vehicle controls and expressed as percentage of the mean of the relative vehicle controls. Compounds which showed a percentage of inhibition against cell proliferation more than 50% are defined as “hit compounds” for each cell line. Vehicle only wells served as intra-plate controls on each plate were uniformly distributed throughout the screen. Background plates containing vehicle only served as inter-plate controls were inserted throughout the screen. The controls were used to calculate the background levels/noises of the assay and to evaluate assay response as well as performance. Performance of the assay was assessed using Z′ factors.

### Quantitative Compounds Screen Assay

Cells were grown to 80% confluence, harvested and aliquoted into 96 well plates at concentrations of 1,000 cells per well in a total volume of 90 µL/well. Hit compounds or vehicle (DMSO) were then loaded to each well to give a final doses range from 50 µmol/L to 0.03 µmol/L (serial four-fold dilutions) in 100 µL total volume. Cells were cultured for 72 hours at 37°C in a 5% CO_2_ atmosphere. Aliquots of 20 µL Alamar-Blue reagent were added directly to each well, the plates were incubated at 37°C for 3 h and the fluorescent signal was measured with an excitation at 530 nm and emission at 590 nm on ZS-2 plate reader. Data were normalized as percentage inhibition relative to vehicle control. IC_50_ values were estimated using a four parameter logistic curve model by SigmaPlot 12.0 (Systat Software, INC).

### Western Blotting

Cells were grown to 70–80% confluence, harvested and aliquoted into 100 mm dishes. Media was removed and replaced by media supplied with different doses of compounds the next day. Dished were incubated for an additional 6 h. Cells were washed with PBS and cell lysates were prepared by scraping cells into RIPA buffer with protease inhibitor and phosphatase inhibitor. The homogenates were centrifuged. The supernatants were transferred to fresh tubes and protein concentrations were determined using the Bio-Rad protein assay (Bio-Rad Laboratories, Inc.) per manufacturer's instructions. Equal amounts of total protein (25 µg) were subjected to electrophoresis (10% tris-HCl, 1.0 mm gels) and transferred to PVDF membranes. Membranes were blocked in TBS containing 0.05% Tween 20 (TBST) and 5% nonfat milk or 5% BSA for 1 hour at room temperature. Primary antibodies were diluted per manufacturer's instructions and incubated in TBST/5% nonfat milk or TBST/5% BSA for 2 h at room temperature or overnight at 4°C. Secondary antibodies were diluted (1∶10,000) and incubated with PVDF membranes for 1 h at room temperature. Proteins were detected by ECL plus western blotting detection system (GE healthcare). The following antibodies were used: anti-phospho-HER2 (Tyr1248), anti-HER2, anti-phospho-AKT (Thr308 or Ser473), anti-AKT, anti-phospho-p42/44MAPK, anti-p42/44MAPK, and anti-β-actin antibodies (Rui Biological Ltd., Shanghai, China).

### Annexin V-FITC assay

Cells were treated with hit compounds for 48 h at 37°C, pooled and washed with PBS for three times, and re-suspended in binding buffer. Cells were then stained with annexin-V-FITC antibody supplied by the Chengdu Medical College Flow-cytometry core facility. Propidium iodide (PI) (final concentration 1 g/ml) was added immediately prior to analysis. Bivariant analysis of FITC fluorescence and PI fluorescence gave different cell populations, where FITC (−) and PI (−) were designated as viable cells, FITC (+) and PI (−) as apoptotic cells, and FITC (+) and PI (+) as late apoptotic or necrotic cells.

### Caspase 3/7 activity assay

Cells were grown to 70–80% confluence, harvested and aliquoted into 96-well plates. Different doses of compounds were added to the plates the next day. Plates were incubated for an additional 48 h at 37°C. Aliquots of Alamar-Blue reagent were added directly to each well, the plates were incubated at 37°C for 3 h and the fluorescent signal was measured with an excitation at 530 nm and emission at 590 nm on ZS-2 plate reader. Then equal volume of caspase 3/7 activity assay reagent (Promega, China) was added to each well and the luminescence signal was measured on ZS-2 plate reader. Data were normalized as luminescence relative to fluorescence.

### *In vivo* efficacy in xenograft models

*In vivo* experiments were carried out under pathogen-free conditions at the animal facility in accordance with the institutional guidelines of the Chengdu Medical College Institutional Animal Care and Use Committee (IACUC). All protocols were reviewed and approved by IACUC and HER2-positive breast cancer cell line MDA-MB-453 cells were resuspended to 2×10^6^ cells/100 µl in PBS and implanted subcutaneously into the flank region of 6–7-week-old female nude mice weighing 18 to 22 gram. When tumors reached 50 to 60 mm^3^ in volume, animals were randomly assigned to 3 groups, receiving either saline (control) or peonidin-3-glucoside (6 mg/kg) and cyaniding-3-glucoside (6 mg/kg) as oral gavage 7 times a week for a total of 25 days. Tumors were measured every 5 days with a caliper and tumor volume (in mm3) was calculated using the following formula: V = 4/3π[(w+l)/4]3, where V = volume (mm3), w = width (mm), l = length (mm). Once the control tumors reached 1000 mm^3^, the animals were euthanized due to ethical requirements. After 25 days treatment, all animals were euthanized using overdosed CO_2_, and the tumor tissues were extracted for immunostaining and weighing. All values are expressed as the mean ± SEM.

### Statistical analysis

*In vitro* data were reported as mean ± SD, each treatment performed in duplicate or triplicate. Data were log-transformed to stabilize variances for proliferation assays. *In vivo* data were reported as mean ± SEM. Values were analyzed using the Student's *t* test or with one-way ANOVA when three groups were present. Statistical significance was considered as *p*≤0.05.

## Results

### High-throughput Screen (HTS) development and assay performance measurements

For optimization of assay performance, the number of cells/well and the length of the incubation period, and the volume of Alamar-Blue reagent were empirically determined (**Table 2**). To identify compounds that might have inhibitory effect against HER2 positive cell proliferation, we used a three-step screen experimental approach to assess the activity of 10,000 natural compound library. First step, a one dose (10 µmol/L) HTS was performed using three HER2-positive breast cancer cell liens including BT474, MDA-MB-453, and HCC1569. Compounds that exhibited precipitation were excluded from the following screen. We have identified 45 compounds that inhibited cell proliferation by more than 50% at 10 µmol/L after a 72-hour incubation. Second step, a one dose (10 µmol/L) screen was performed using three HER2-negative breast cancer cell lines including MCF-7, SUM190, and MDA-MB-231 using hit compounds from first step. The assay steps were summarized in **Table 3**. Controls present in each assay plate consisted of 0.2% DMSO (vehicle control) or afatinib (positive control). Combined with the result from first two steps, we have identified 8 compounds that selectively inhibited HER2-positive breast cancer cell proliferation by more than 50% at 10 µmol/L after a 72-hour incubation (step 1) compare to no anti-proliferation activity against HER2-negative breast cancer cells (step 2). The protocol of the preliminary screen study is summarized in **Table 3**. A total of 543 plates were screened. The assay performed well over the entire course of the screen as demonstrated by the average of Z′ values (0.58) [9].

**Table 2:** Screen assay protocol for step 1 and 2.

Step	Event	Parameter	Description	Notes
**1**	Add reagent	90 µL	1,000 cells/well	
**2**	Add library compounds or DMSO	10 µL	10 µmol/L	
**3**	Incubate	72 hrs	5% CO_2_/37°C incubation	
**4**	Add reagent	20 µl	Alamar-blue reagents	
**5**	Incubate	4 hrs	5% CO_2_/37°C incubation	
**6**	Read	Fluorescence	530 nm Ex/590 nm Em, gain = 60.	Instrument:Zs-2 plate reader

Plates lidded until read.

**Table 3:** FP assay performance and post-screen analysis summary.

Category	Parameters	Descriptions
Assay	Nature of the assay	Cell-based proliferation assay
	Assay strategy	Detection of cell proliferation using Alamar-blue reagents
	Reagents and sources	See materials and Methods
	Assay protocol	Key steps outlined in Table **2**
Library screened	Nature of the library	Fractions derived from natural Chinese medicine
	Size of the library	10,000 natural compounds arrayed in 96-well plates as single compounds at 10 mmol/L in DMSO
	Source	Pharmanic, China
	Quality control	All compounds assured by the company as >98% pure on HPLC with QC data
	Concentration tested	10 µmol/L in 0.2% DMSO (v/v) for step 1 and 2. Serial dilutions for step 3.
	Format	96-well plate
	Plate controls	Positive control: afatinib; negative control: 0.2% DMSO
	Plate number and duration	Step 1: 540 plates, 5 monthStep 2: 3 plates, 2 weeksStep 3: 10 plates, 2 weeks
	Output, detector, analysis software	ZS-2 detector; Fixed endpoint; Fluorescence value, SigmaPlot
	Normalization	% inhibition = 100 x (sample result – average of vehicle treated control)/average of vehicle treated control
	Performance	Z′ = 0.58
Post-screen analysis	Selection of actives	Actives were selected from the primary screen using a threshold of better than 50% inhibition
	Retesting of initial actives	Original samples retested using screening assay condition; compounds with triplicated activity tested in dose-response mode (8 half dilutions)
	List of validated compounds	Table **4**

### Hit compounds inhibit the growth of HER2-postive cancer cell lines

**Figure 1:**
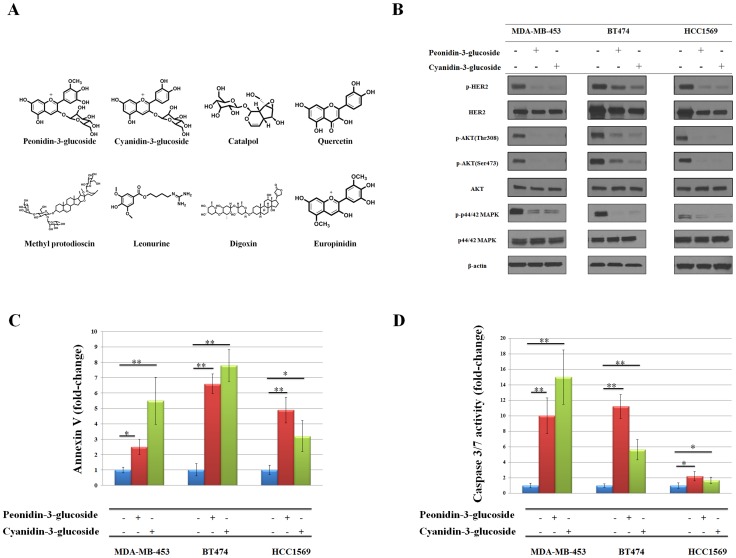
*In vitro* antitumorigenic activity of peonidin-3-glucoside or cyaniding-3-glucoside. (A). Structures of hit compounds. (B) Effect of peonidin-3-glucoside and cyanidin-3-glucoside on HER2 and downstream signaling. BT474, MDA-MB-453, and HCC1569 cells were treated with or without peonidin-3-glucoside or cyanidin-3-glucoside for 6 h and whole-cell extracts were analyzed by western blotting with the indicated antibodies. (C) Peonidin-3-glucoside and cyanidin-3-glucoside treatments induce apoptosis in MDA-MB-453, BT474, and HCC1569 cells. Cells were treated with or without peonidin-3-glucoside or cyanidin-3-glucoside for 48 h and annexin V positive cells were counted using flow cytometry. (D) Peonidin-3-glucoside and cyanidin-3-glucoside treatments increase caspase 3/7 activity in MDA-MB-453, BT474, and HCC1569 cells. Cells were treated with or without peonidin-3-glucoside or cyanidin-3-glucoside for 48 h and cell viability and caspase 3/7 activity was measured as per the manufacturer's instructions. Data were normalized as caspase 3/7 activity divided by cell viability. Data represent mean ± SEM. *p<0.05, **p<0.01.

Third step, hit drugs that only inhibit HER2-positive cell line proliferation were chosen for a quantitative screening to generate IC_50_ values as described in the Materials and Methods section. The eight candidates's structure were summarized in **Figure 1A** and were verified and their IC_50_ values were confirmed (**Table 4**). All eight compounds showed a dose-dependent inhibition of BT474, HCC1569, and MDA-MB-453 cell proliferation with less than 10 µmol/L IC_50_ values (**Table 4**). Based on the activities and safety profiles of the hit compounds, we prioritized to validate the top two hits: peonidin-3-glucoside and cyaniding-3-glucoside.

**Table 4:** Summary of hit compounds.

Number	Compound	Derived from	IC_50_ (µM)		
			BT474	HCC1569	MDA-MB-453
**1**	Peonidin-3-glucoside	Black rice	5.2±0.8	4.3±0.6	1.2±0.5
**2**	Cyanidin-3-glucoside	Black rice	1.8±0.5	2.6±0.8	1.5±0.3
**3**	Catalpol	*Rehmannia glutinosa*	2.4±0.4	1.9±0.7	2.7±0.6
**4**	Quercetin	*Cuscuta chinensis Lam*	4.4±0.3	4.9±1.1	4.0±0.6
**5**	Methyl protodioscin	Cochinchinese Asparagus Root	3.5±0.6	3.2±0.8	4.2±0.5
**6**	Leonurine	*Rehmannia glutinosa*	3.6±0.8	4.5±0.9	4.6±0.8
**7**	Digoxin	*Digitalis lanata*	4.2±0.7	3.6±0.8	4.7±0.4
**8**	Europinidin	*Plumbago zeylanica*	5.8±1.5	7.0±1.8	8.0±1.2

### Hit compounds inhibit HER2 in HER2-postive cancer cell lines

We first determined the phosphorylation status of the HER2 protein and its downstream mediator AKT to further confirm the anti-proliferation activities of the hit drugs are due to selective inhibition of HER2 protein. To evaluate the response of the cell lines to hit drugs, BT474, MDA-MB-453 and HCC1569 cells were treated with drugs (10 µmol/L) for 6 h. Western blotting show that both peonidin-3-glucoside and cyanidin-3-glucoside significantly reduce the phospho-HER2, phospho-AKTs, and phopspho-p44/42MAPK levels compared to control cells (**Fig. 1B**).

### Hit compounds induce apoptosis in HER2-postive cancer cell lines

To further confirm the hit compounds activity, we performed an Annexin V staining assay and a caspase 3/7 activity assay to detect apoptosis events. Our result show in **Figure 1C&D** supported the cell viability assay results (**Table 4**). Three HER2-positive breast cancer cell lines, MDA-MB-453, BT474, and HCC1569 were treated with or without hit compounds (5 µmol/L) for 48 h. Both peonidin-3-glucoside and cyaniding-3-glucoside significantly induced apoptosis in all tested lines compared to their controls.

### Antitumor activity of hit compounds *in vivo*

**Figure 2:**
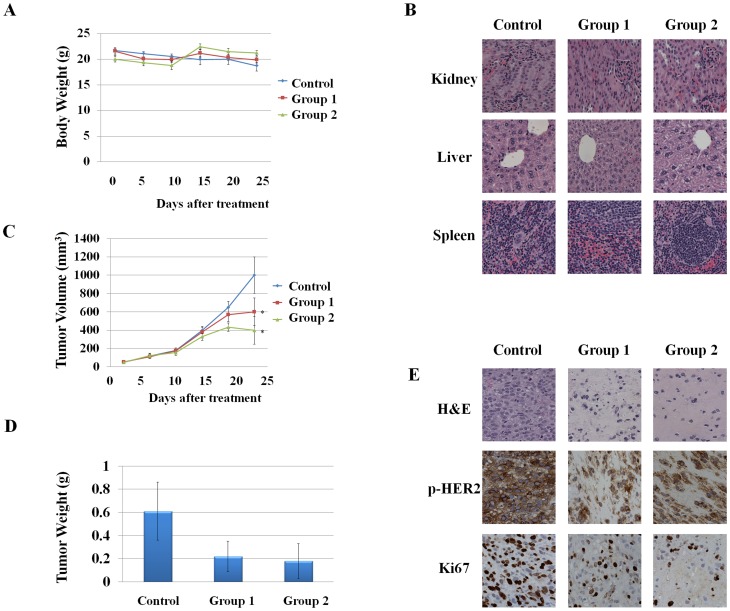
Antitumorigenic activity of peonidin-3-glucoside or cyaniding-3-glucoside in xenografted nude mice. (A) Body weight of animals. (B) H&E staining for kidney, liver, and spleen. (C and D) Effects on tumor volume and tumor weight. Nude mice bearing MDA-MB-453 cells as xenografts were treated with control (saline), or group 1 (peonidin-3-glucoside (6 mg/kg/day)) or group 2 (cyaniding-3-glucoside (6 mg/kg/day)). Values are means ± SE (n = 10). *P<0.05. (E) H & E staining, expression of phospho-HER2 and Ki67. Control: saline; Group 1: peonidin-3-glucoside (6 mg/kg/day); group 2: cyaniding-3-glucoside (6 mg/kg/day).

To assess the antitumor effect of peonidin-3-glucoside and cyaniding-3-glucoside in a xenograft model of HER2-positive breast cancer, MDA-MB-453 cells were used in female nude mice. After 25 days of peonidin-3-glucoside (6 mg/kg) and cyaniding-3-glucoside (6 mg/kg) treatment, animal weight stayed stable during the treatments in each group (**Fig. 2A**). Animals treated with drugs did not exhibit signs of organ damage (**Fig. 2B**). Tumor growth rates were significantly different from control group (**Fig. 2C**). Accordingly, final tumor volumes (**Fig. 2C**) and weights (**Fig. 2D**) were suppressed in the peonidin-3-glucoside (6 mg/kg) and cyaniding-3-glucoside groups compared to the control group. This suppression was accompanied by decreased phospo-HER2 levels and decreased proliferation marker Ki67 levels (**Fig. 2E**).

## Discussion

Identification and characterization of new pharmacological activities from existing Chinese Traditional Medicines represents an effective way to accelerate the translation of discoveries at the bench to clinical applications partially due to the known clinical effects of the drugs. To date, a number of interesting hits have been identified.

In the present study, we performed a three-step screen for searching anti-HER2 positive breast cancer agents. In step 1, we screened the entire 10,000 Chinese Traditional Medicine extracts library against three HER2-positive cell lines. In step 2, we screened the hit compounds from step 1 against three HER2-negative cell lines. In step 3, we generated the IC_50_ values for the hit compounds which selectively inhibit HER2-positive cell proliferation.

Peonidin-3-glucoside and cyaniding-3-glucoside are anthocyanin pigments in colored rice cultivars [10]. They have been reported to have anti-cancer properties [11]-[16]. Anthocyanins have been used as medicine or supplements for many years and it has been reported that depending on the nutrition habits, the daily intake of anthocyanins in humans could be up to 150 mg/day [14]. Catalpol is an iridoid glucoside and exhibits anti-inflammatory and anti-diabetes activities [17]–[19]. Quercetin is a natural flavonoid widely distributed in plants that acts as a neuroprotective, anti-cancer, anti-ROS formation agent [6], [20]–[23]. Methyl protodioscin is one of the main bioactive extracts from the Traditional Chinese Medicine *Dioscorea collettii var hypoglauca*. Studies show that methyl protodioscin has anti-proliferative effects on cancer cells due to the induction of cell cycle arrest and/or apoptosis [13], [24]–[29]. Leonurine exerted cardioprotective, antioxidant, and anti-inflammation properties [30]–[33]. Digoxin is a purified cardiac glycoside extracted from *Digitalis lanata*. Digoxin is widely used in the treatment of various heart conditions including atrial fibrillation, atrial flutter and heart failure that cannot be controlled by other medication [34]–[36]. Digoxin is a very potent Na+/K+ ATPase pump inhibitor with a very narrow therapeutic index. Therefore extra cautious is needed when use this drug [35], [37]. Europinidin is a water soluble, bluish red plant dye derived from *Plumbago zeylanica*. There is no citable reference for this compound regarding its activities.

We prioritized to study peonidin-3-glucoside and cyaniding-3-glucoside and showed significant anti-tumor activity *in vitro*. To further evaluate the efficacy of these two compounds, we conducted *in vivo* anti-tumor experiments using immune-compromised mice model. It has been reported that depending on the nutrition habits, the daily intake of anthocyanins in humans could be up to 150 mg/day [14]. We conservatively choose the safe human dose as 30 mg/day to convert the dosage to the mouse equivalent does using the recommended conversion of animal doses to human equivalent does based on body surface area by US Food and Drug Administration (2005). We calculated the equivalent doses of human (30 mg/day) is equal to 6 mg/kg/day to mouse assuming the human body weight is 60 kg and the mouse body weight is 0.020 kg. The formula listed below has been used:

Animal equivalent dose  = (30 mg/60 kg) x 12.3

Both peonidin-3-glucoside and cyaniding-3-glucoside showed significant anti-tumor activity *in vivo*, however, there is no significant difference between the two treatments.

## Conclusions

In this study, we screened a 10,000 Chinese Medicine extracts library against three representative HER2-positive breast cancer cell lines for anti-proliferation drugs and identified 45 hits in step 1. In step 2, the hits identified from step 1 were profiled against three representative HER2-negative breast cancer cell lines. We identified eight compounds as selective anti-proliferation agents of HER2-positive breast cancer cells. In step 3, those eight compounds have been validated to generate IC_50_ values. Very interestingly, there are two compounds, peonidin-3-glucoside and cyaniding-3-glucoside were extracted from the same source (Black rice). To initiate the validation, we also tested the ability of these drugs to induce the early apoptosis and further confirmed the anti-proliferation abilities. Studies using nude mice bearing MDA-MB-453 cells confirmed the antitumorgenic effects of peonidin-3-glucoside and cyaniding-3-glucoside treatments *in vivo*. In conclusion, the discovery of HER2 selective anti-proliferation of breast cancer cells by Chinese Traditional Medicines have important implications in the development of these drugs as new anti-breast cancer agents.
